# Using MOEA with Redistribution and Consensus Branches to Infer Phylogenies

**DOI:** 10.3390/ijms19010062

**Published:** 2017-12-26

**Authors:** Xiaoping Min, Mouzhao Zhang, Sisi Yuan, Shengxiang Ge, Xiangrong Liu, Xiangxiang Zeng, Ningshao Xia

**Affiliations:** 1 School of Information Science and Technology, Xiamen University, Xiamen 361102, China; mxp@xmu.edu.cn (X.M.); zhangmouzhao@stu.xmu.edu.cn (M.Z.); sisiyuan.xmu@gmail.com (S.Y.); xrliu@xmu.edu.cn (X.L.); 2 National Institute of Diagnostics and Vaccine Development in Infectious Diseases, Xiamen University, Xiamen 361102, China; sxge@xmu.edu.cn (S.G.); nsxia@xmu.edu.cn (N.X.)

**Keywords:** many-objective optimization, phylogenies, consensus, genetic algorithm

## Abstract

In recent years, to infer phylogenies, which are NP-hard problems, more and more research has focused on using metaheuristics. Maximum Parsimony and Maximum Likelihood are two effective ways to conduct inference. Based on these methods, which can also be considered as the optimal criteria for phylogenies, various kinds of multi-objective metaheuristics have been used to reconstruct phylogenies. However, combining these two time-consuming methods results in those multi-objective metaheuristics being slower than a single objective. Therefore, we propose a novel, multi-objective optimization algorithm, MOEA-RC, to accelerate the processes of rebuilding phylogenies using structural information of elites in current populations. We compare MOEA-RC with two representative multi-objective algorithms, MOEA/D and NAGA-II, and a non-consensus version of MOEA-RC on three real-world datasets. The result is, within a given number of iterations, MOEA-RC achieves better solutions than the other algorithms.

## 1. Introduction

With the rapid growth of the number and size of biological molecular sequences discovered, inferring phylogenetic tree that describes evolutionary relationship between given molecular sequences is getting more important and harder in field of bioinformatics. The computational methods for inferring a phylogenetic tree have been split into the following three categories: (1) distance-matrix methods, such as NJ [1] and BIONJ [2], generate trees stepwise using genetic distance information obtained from multiple sequence alignments under the assumption that the tree with the smallest sum of branch lengths is the best; (2) Maximum Parsimony (MP) [3] calculates and minimizes the total amounts of variation of phylogenetic trees on the hypothesis that, the fewer mutation events occur, the more authentic they are; and (3) Maximum Likelihood (ML) [3], which estimates phylogenetic trees based on alternative evolutionary Markov models, such as JC69 [4], HKY85 [5], TN93 [6] and GTR [7] that describe the rates at which one nucleotide replaces another. One tree, having a high ML value, indicates the genetic relations between the sequences described in the tree are more realistic.

Inferring phylogenetic tree can be treated as single objective optimization problem or multi-objective optimization problem (MOP) [8] which we discuss later. When it comes to single objective problem, the process of inferring phylogenetic tree is guided by one of criteria described above, such as ML or MP. Phylogenetic trees about same molecular sequences are always comparable under single criterion. As explained by Lemmon [9], to obtain the optimal solution, using any one of these two methods, we must rebuild all possible phylogenetic trees from one set of sequences, and that becomes difficult or impossible as the number of sequences increases. It becomes a NP-hard problem that cannot be solved in polynomial time [10]. Thus, to save computational time, a more practical way to solve this kind of problem is to find an approximate solution which is near optimal solution, instead of a real optimal solution. The following are three strategies that can be used to find an approximate solution: (1) Hill-climbing, i.e., repeating branch swapping and parameter optimizing (including branch length optimizing) until a better solution cannot be obtained after several iterations [11]; (2) divide-and-conquer, such as the quartet puzzling method [12]; and (3) the relative effective way is to use metaheuristics, such as genetic algorithms [13,14,15], particle swarm [16], etc. Besides these algorithms, there is much software, available online, based on a single criterion. For example, TNT [17] and PHYLIP [11] are based on MP. RAxML [18], IQPNNI [19], MrBays [20], Garli [21], and MetaPIGA [22] are based on ML. TNT implements different heuristic, such as sectorial and tree fusing, to address the inference of maximum parsimony trees.

Compared to single objective optimization problem, MOP is more complex, which deals with two or more objective functions. In MOP, no solution can optimize all objective functions simultaneously since objective functions are not all compatible. Instead of one optimal solution, each MOP has a Pareto Front (PF) that is composed by optimal solutions. The Pareto dominance concept is used to compare two solutions. A solution x dominates a solution y if x is not worse than y in all objectives and if it is better for at least one. Those optimal solutions in PF are non-dominated. In solving MOP, we search some Pareto-optimal solutions that must be uniformly approaching to PF. Multi-objective evolutionary algorithms (MOEAs) is a common way to deal with MOP.

Since two criteria can be the objective functions of MOP and the results of these two criteria are conflicting [23,24,25], more and more studies [26] focus on considering the inferring of phylogenetic trees as a MOP. Besides inferring phylogenetic trees, actually, a wide range of biological problems have been regarded as MOP [27]. It has been testified [28] that multi-objective genetic algorithms will outperform single-objective genetic algorithms because multi-objective genetic algorithms are more likely to escape from local optima. Based on a well-known MOEA called NSGA-II [29], PhyloMOEA [8] was proposed for phylogenetic inference and it performs well in both ML and MP.

For any MOEAs applied to inferring phylogenetic tree, they must evaluate all trees at each generation. If ML and MP were chosen as objective functions, computing the result of these two functions for all trees can be very time-consuming. In general, two kinds of method can be used to enhance algorithm’s time efficiency: (1) parallelizing algorithm [16,30,31]; and (2) improving algorithm to achieve fine convergence in fewer generations. Our work is focused on the second one.

Lemmon used the inter-population consensus information to enhance accuracy and speed of genetic algorithm [9] which is used to infer phylogenetic tree. In this context, consensus information refers to the consensus branches, obtaining from several elitist phylogenetic trees that can be treated as common topological features contributing much to the evaluation result of those elitists. Under the assumption that those consensus branches are correct, Lemmon’s algorithm protects these consensus branches from any topological mutation. With the help of consensus information, the algorithm avoids many random searches in solution space and converges in fewer generations. However, only one criterion, ML, has been considered in Lemmon’s algorithm. Therefore, in this paper, to use consensus information to speedup MOP in inferring phylogenetic tree, we designed new MOEA—Multi-Objective Evolutionary Algorithm using Redistribution and Consensus (MOEA-RC)—that fits consensus branches well.

## 2. Materials and Methods

In this paper, two classical MOEAs were chosen to try to fit consensus: MOEA/D [32] and NSGA-II [29]. The main procedure for NSGA-II is as follows: In every generation, NSGA-II generates new offspring by a genetic operation and then splits the population, including parents and offspring, into several subsets called non-dominated front that are marked as different fitness levels by a domination relationship. Then, the better subsets are chosen as the parents of the next generation. Because consensus must be computed from several solutions, consensus may be obtained from NSGA-II’s non-dominated front. As we view inferring phylogenetic tree as MOP, no tree can optimize the two objectives simultaneously, which is the same as consensus that represents tree’s topological characteristics. Different consensuses contribute to different objective functions in different degrees. Those solutions in NSGA-II’s non-dominated front represent diverse topological characteristics and, usually, are good at one objective function but inferior at another one. Therefore, we cannot compute right consensus from non-dominated front.

MOEA/D [32] is a MOEA based on decomposition that, via an aggregation function, separates the MOP into several smaller, single objective sub-problems and then, using a common evolutionary algorithm, solves the sub-problems. In MOEA/D [32], weight vector is used to aggregates multiple objective functions to one by aggregation function; therefore, trees owning the same weight vector have the similar topological characteristics. However, this is not suited for computing consensus, because every weight vector corresponds to only one solution, while consensus needs to be computed from more than one solution.

To fit the concept of consensus into MOEAs, we designed a new MOEA called MOEA-RC to infer phylogenetic tree. The RC in the name of MOEA-RC is an abbreviation of redistribution and consensus that are core operations in this new MOEA. The range of values of the two objectives, MP and ML, is different in most cases. Consequently, the following normalization must be applied when evaluating solutions:

(1) $${\bar{f}}_{i}=\frac{f_{i}-z_{i}^{*}}{z_{\mathrm{nad}}^{i}-z_{i}^{*}}$$

${\bar{f}}_{i}$ is the value after normalize ith function in m objective functions; $z^{*}=\left(z_{1}^{*},\ldots ,z_{m}^{*}\right)$ is the reference point; $z^{\mathrm{nad}}=\left(z_{1}^{\mathrm{nad}},\ldots ,z_{m}^{\mathrm{nad}}\right)$ is the nadir point; and $f_{i}$ is the original ith objective function value. $z^{*}$ and $z^{\mathrm{nad}}$ will be dynamically obtained at runtime. We check the best value and worst value for every objective function and update the value of $z^{*}$ and $z^{\mathrm{nad}}$ at each generation.

### 2.1. Selection Operator-Redistribution

**Figure 1:**
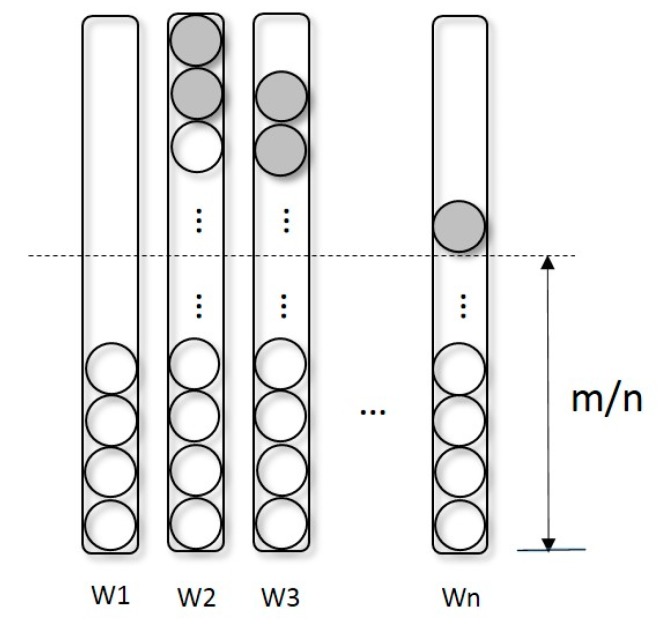
Illustration of redistribution. Rectangles marked W1–Wn are populations for different weight vectors. m is the number of defaults of all the individuals. The number of individual cycles is denoted by 2 × m. m number of grey cycles are eliminated in each iteration of redistribution.

Our proposed algorithm is composed of two critical components: redistribution and consensus. Redistribution, which selects survivals and offers a reasonable condition for computing a consensus, is described as follows. Let P, having size m, denote the parent populations, which survived from the last selection of the algorithm. S, also having size m, denotes the offspring generated by the genetic operation from P. C is the union of P and S. As shown in Figure 1, C is the input of redistribution. In the solution sets, each solution is distributed to the sub-population which most closely related with this solution. The number of sets, defined by the user, is identical to the number of weights. The method for measuring relative degree is expressed by Equation (2). ℛ denotes relative degree; ${\bar{f}}_{1}$ and ${\bar{f}}_{2}$ are normalized values of objective functions of individuals; and $w_{i}^{1}$ is the first dimension value of the ith weight vector.

(2) $$Minimize\;ℛ=\left|\frac{{\bar{f}}_{1}}{{\bar{f}}_{1}+{\bar{f}}_{2}}-w_{i}^{1}\right|\;\mathrm{subject}\;\mathrm{to}\;i\in \left\{1,\;2,\;.\;.\;,\;n\right\}$$

As mentioned above, redistribution is responsible for selecting survivors from the population. Therefore, after the above operation, we choose the solution that can survive. The size of C is 2 × m; thus, the number of solutions, m, in C are eliminated. Ideally, after selection, each solution set has the same number of solutions as the other solution sets. To approach the ideal, after sorting the solutions according to their fitness computed by the Weighted Sum (WS) approach (Equation (3)), we eliminate the worst solutions in those sets with size exceeding m/n.

(3) $$G^{\mathrm{ws}}(x|w_{i})=\overset{n}{\underset{j=1}{\sum }}w_{i}^{j}f_{j}$$

The grey solutions in Figure 1 are eliminated; only those solutions outside the dashed line are possibly eliminated. The reason for doing this is to keep as sufficient a number of solutions as possible to improve the validity of the consensus generated in the next step. On the other hand, retaining those few population solutions enhances the distribution and variety of the populations.

### 2.2. Consensus

After selection, we compute the consensus for those solution sets that have a different search direction. For example, for solution set T, compute the consensus from T and T’s two neighboring sets, which depend on the distance of their corresponding weight vectors. We implemented the more efficient method to compute majority rule (+) consensus described in the work [33]. However, differing from the work [33], instead of consensus trees, we compute the consensus branches of trees. Those consensus branches are viewed as the most correct branches in the current population. We try to protect those branches in any subsequent operation, such as crossover and mutation, that would change the topology of the trees.

Three sets are used to compute the consensus for one set for the following reason: Those solutions that are involved in computing a consensus are regarded as elites in the population. Moreover, if we choose all the solutions in the population to compute consensus branches, the result would be that all the solutions in one direction are exactly the same after a few generations because of consensus. Therefore, to compute a consensus, we choose prior solutions, rather than all the solutions in the set. However, the elites in one solution are too few to result in a correct consensus. We fix this problem by using the idea of the neighbor in MOEA/D [32]. The neighbors of one weight vector are seen as being in the same direction to some degree. Thus, instead of all the solutions in T, we use the few best solutions in the three sets neighboring T.

### 2.3. MOEA-RC

The step-by-step procedure for MOEA-RC is as follows:

Step 1. Population initiation: Two options can be chosen to initialize population: (1) using molecular sequences, which have been pre-processed, such as alignment, to randomly build N phylogenetic trees; and (2) using user given trees.
Step 2. Evaluation: Evaluate trees by MP and ML and update reference point at the same time.
Step 3. Redistribution: As described in Section 2.1, separate the population into several sub-populations according to Equation (2) and Sort trees in each sub-population by their fitness computed by Equation (3).
Step 4. Computation of consensus: Generate consensus branches for each sub-population as described above.
Step 5. Generation of offspring: Randomly select two individuals from the current population and do a crossover operation to generate two new solutions. Many crossover operators are available in the literature (Lewis 1998 [13]; Congdon 2002 [34]). We chose Prune–Delete–Graft (PDG), which is described by Lewis (1998) [13], because it has been successfully applied under different criteria. Then, use the nearest neighbor interchange (NNI) [35] to mutate the two new solutions. Repeat this step until N new solutions have been generated.
Step 6. Merging: Merge the offspring with the current population.
Step 7. Repeat: Return to Step 2 and repeat until the stop condition is reached. The stop condition in this paper is the maximum number of evaluation times set by the user. The stop condition in this paper is a given specific number of generations.

The major computational costs in MOEA-RC are Steps 2–5. It takes *O*(N) time to evaluate population in Step 2. In Step 3, every tree in population needs *O*(w) time to compute Equations (2) and (3). Thus, the complexity of this step is *O*(w×N). For Step 4, as reported by Jansson [33], given an input of k phylogenetic trees with identical leaf label sets and n leaves each, we can obtain consensus branches in *O*(k×n) time. Because we need w sets of consensus branches and the upper bound of k in this context is 3 N/w, Step 5 takes *O*(n×N) time.

## 3. Results

To validate our proposed algorithm, we conducted a series of experiments. Firstly, we compare our algorithm with the two most popular and representative algorithms, MOEA/D and NSGA-II, on three real-world datasets (See Table 1) using random starting trees as initial population. Likewise, another algorithm (MOEA-R) without a computing consensus that could testify to the efficacy of the consensus was used in the experiments. Secondly, we compare our algorithm with PhyloMOEA, MOEA/D and NSGA-II using same starting trees, which were generated by bootstrap analysis before running experiments, as initial population for the sake of fairness. The reason for using given tree is the same as mentioned in [8]: random starting trees are poor estimations of ML and MP. Our proposal algorithm was also compared with several single-criterion phylogenetic software. All experiments in this paper were independently run ten times on the same server (Intel(R) Xeon(R) E5-2630 v3 CPU at 2.4GHz and 64G RAM, PowerEdge R730, Dell Inc., Xiamen, China). The server’s operating system is Ubuntu 5.4.0-6. The common parameters for all evolutionary algorithms in this experiment are listed in Table 2. Those experiments on same dataset used same substitution model, GTR + GAMMA, with same specific parameters generated by RAxML. The aggregation function used by MOEA/D in this paper is Tchebycheff [32].

**Figure 2:**
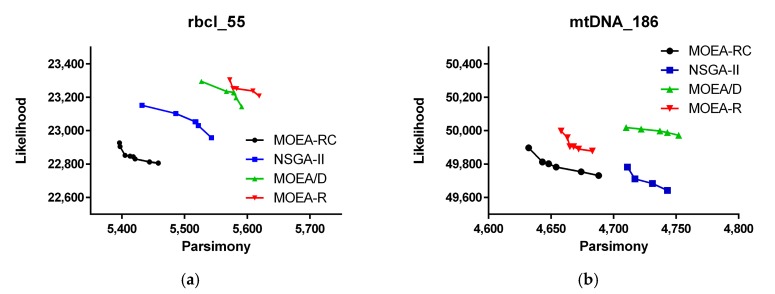

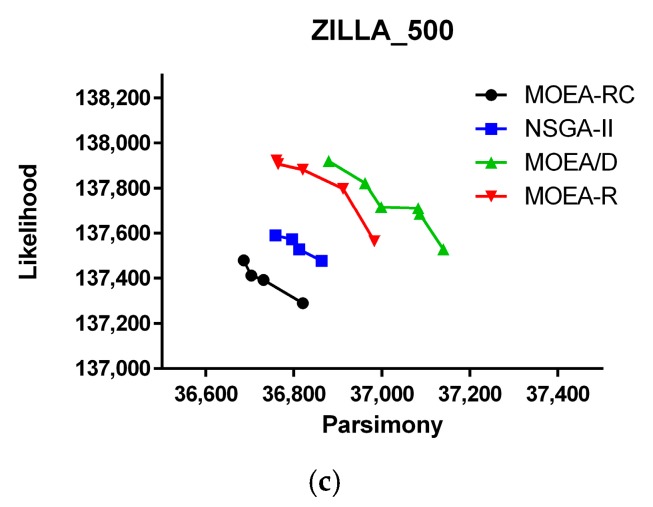
Pareto Front (PF) of the four algorithms in the three datasets at 1000 evaluation: (**a**) performances on rbcl_55; (**b**) performances on mtDNA_186; and (**c**) performances on ZILLA_500.

**Figure 3:**
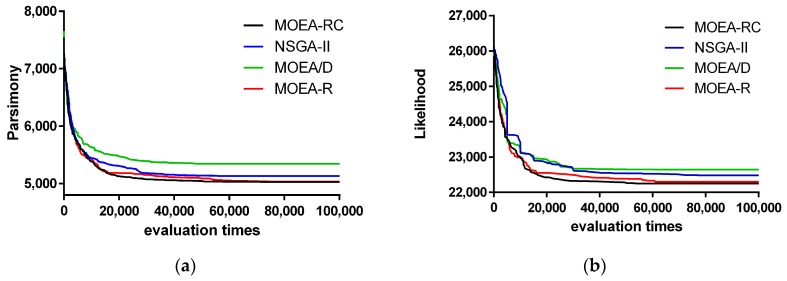
Change of ML and MP of the algorithms with various multiples of evaluation on rbcl_55: (**a**) Maximum Parsimony; and (**b**) Maximum Likelihood.

**Figure 4:**
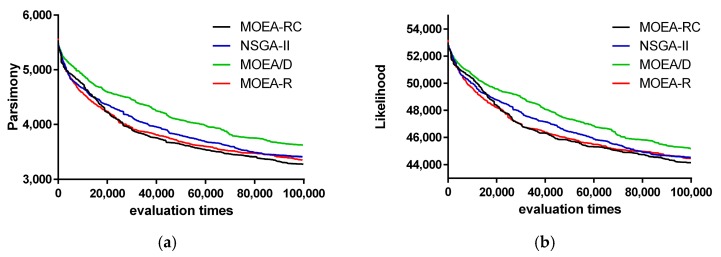
Change of MP and ML of the algorithms with various multiples of evaluation on mtDNA_186: (**a**) Maximum Parsimony; and (**b**) Maximum Likelihood.

The Pareto Front (PF) of the four algorithms in the three datasets at a 100 evaluation is shown in Figure 2, Figure 3 and Figure 4. Figure 2, Figure 3 and Figure 4 show the change of MP and ML of the algorithms with various multiples of evaluation on rbcl_55 and mtDNA_186. In these figures, the results are composed by the best one time of 10 times independent run for each algorithm and the value of ML have been multiplied by −1 so that we can simply minimize all objective functions.

As shown in Figure 2, MOEA-RC has a better PF than the three other algorithms at 100 the evaluation. The non-consensus version of MOEA-RC, MOEA-R, achieved a performance about the same as that for NSGA-II and MOEA/D. Specifically, MOEA-R performs better than MOEA/D in mtDNA_186 and ZILLA_500. In dataset mtDNA, the PF of MOEA-R and NSGA-II are mutually non-dominated. To a certain degree, the advantage of MOEA-RC compared with MOEA-R demonstrates the efficacy of consensus.

The results (Figure 3) show the convergence of those algorithms. Compared with the other three algorithms, MOEA-RC has better convergence. Although the curves of MOEA-RC and MOEA-R are very close, MOEA-RC is better than MOEA-R; the high quality of MOEA-RC is more obvious at 20,000 to 60,000 evaluations. In particular, the shape of the curve of MOEA-RC is smoother than the shape of the curve of MOEA-R, illustrating that consensus not only accelerates convergence, but also enhances the stability of the search procedure. On the other hand, the two graphs in Figure 3 also show that MOEA-RC is superior to MOEA/D and NSGA-II. Because either MP or ML and the result of convergence were not good enough, MOEA/D almost ceased improving from 40,000 evaluation. NSGA-II, although better than MOEA/D, still ceased earlier than MOEA-RC. In Figure 4, MOEA-RC also has better result than other three algorithms.

We conclude that consensus can help MOEAs converge in less generations. In fact, as we know, the complexity of NSGA-II is *O*(N^2^), thus MOEA-RC will not take more time to run than NSGA-II while N ≥ n. Table 3 shows MP, ML and execution time of MOEA-RC, MOEA/D, NSGA-II and PhyloMOEA on three datasets. In this table, MP and ML are the best results of 10 independent runs of each program and run time is average run time of 10 independent runs. As we can see, MOEA-RC has the best result among these algorithms. In terms of run time, when the number of taxon (molecular sequence) is smaller than the size of the population, the run time of MOEA-RC is less than the others. This advantage reduces when the number of taxon increases. However, MOEA-RC’s actual run time is still not longer than the others.

In addition, we compare our proposal algorithm with DNAPARS, RAxML and MEGA 7 (Table 4). DNAPARS is one program of PHYLIP that is phylogeny inference package computer programs for inferring phylogenies. RAxML is one of the state-of-the-art tools for Maximum-likelihood based phylogenetic inference. MEGA 7, which is sophisticated software suite for analyzing DNA and protein sequence, can be used to infer maximum parsimony trees and maximum likelihood trees. The configuration of DNAPARS is as follows, search option is more thorough search, the number of trees to save is 100. RAxML’s substitution model setting is GTRGAMMA and starting trees are same as MOEA-RC. The substitution model and starting trees of MEGA 7 are same as RAxML. The other settings of these software packages are default. Because the evaluation method is diverse in different software, we reevaluated the optimal trees generated by all algorithm and software using Bio++ and Phylogenetic Likelihood Library (PPL). In Table 4, we can find MOEA-RC is much better than the other software in terms of likelihood. However, for parsimony, it is only better for one dataset (rbcl_55).

## 4. Conclusions

In this paper, we proposed a novel MOEA called MOEA-RC for phylogenetic inference. MOEA-RC can get better convergence in shorter time using consensus information of phylogenetic trees. We did comparisons of effectivity and efficiency between several state-of-the-art algorithms and tools for inferring phylogenetic tree. The experiments shows that, with help of consensus information, our new algorithm has better convergence to some degree. However, due to weak crossover and mutation operator, the final result of the algorithm is not ideal. Our algorithm underperforms MEGA7 and DNAPARS on two datasets in terms of maximum parsimony. Therefore, in future work, we will find a more reasonable genetic operator to apply consensus information.

## Figures and Tables

**Table 1:** Three datasets for experimentation.

Dataset	Number of Sequence	Nucleotides per Sequence	Data Description
rbcl_55	55	1314	rbcl plastid gene [13]
mtDNA_186	186	16,608	Human mitochondrial DNA [36]
ZILLA_500	500	759	500 rbcL sequences from plant plastids [37]

**Table 2:** Common parameters of the algorithms in the experiments.

Parameter	Value
Population size	100
Generations	100
Selection method	Binary tournament [9]
Crossover method	Prune-Delete-Graft [13]
Crossover probability	0.8
Mutation method	NNI [35]
Mutation probability	0.2
Substitution model	GTR [7]

**Table 3:** The comparison of several multi-objective algorithms on three real-world datasets. The ML and MP in this table is the best value after 100 generations of each algorithm. The mark of (+) means the value is better than the other algorithms.

Algorithm	Metrics	Dataset		
		rbcl_55	mtDNA_186	ZILLA_500
MOEA-RC	ML	−22,156.4 (+)	−39,927.6 (+)	−84,633.1 (+)
	MP	4977 (+)	2460 (+)	17,186 (+)
	Run time	1059.80	66,215.13	13,016.22
MOEA/D	ML	−22,169.6	−39,937.5	−84,715.9
	MP	4979	2461	17,194
	Run time	1302.64	65,620.20	13,082.54
NSGA-II	ML	−22,193.3	−39,942.5	−84,719.4
	MP	4979	2463	17,192
	Run time	1287.07	66,010.94	12,812.62
PhyloMOEA	ML	−2200.1	−39,938.2	−84,704.6
	MP	4982	2461	17,191
	Run time	2163.4	>24 h	>24 h
MOEA-R	ML	−22,244.7	−39,984.6	−84,714.6
	MP	4979	2464	17,196
	Run time	1061.13	65,174.37	12,442.53

**Table 4:** The comparison of MOEA-RC and several phylogenetic software packages on three real-world datasets. The mark of (+) means the value is better than the other algorithms.

Metrics	Algorithm	Dataset		
		rbcl_55	mtDNA_186	ZILLA_500
MP	MOEA-RC	4977 (+)	2460	17,186
	DNAPARS	4984	2438 (+)	17,055 (+)
	MEGA 7	4978	2457	17,077
ML	MOEA-RC	−22,156.4 (+)	−39,927.6 (+)	−84,633.1 (+)
	Raxml	−22,188.2	−40,921.0	−95,577.0
	MEGA 7	−22,205.2	−40,957.0	−95,834.0
